# Complete Heart Block with Normal Bundle of His

**Published:** 1913-03

**Authors:** 


Complete Heart Block With Normal Bundle of His. Wm. Pepper and J. H. Austin, *Am. Jour. Med. Sci.*, page 716, vol. 143, 1912. The case was probably syphilitic but salvarsan had no effect. There was some encroachment on the right branch of the bundle of His by fat and fibrous tissue. (Note—While the vagi were not examined and it is questionable whether the "encroachment," without atrophy, is significant, the case is of value as indicating that the general impression that heart block is always due to a lesion of the bundle of His, is not correct).

